# The Impact of Comorbidity and Age on the Risk of Hospitalization and Mortality in Patients with Previous COVID-19 Infection—Based on Nationwide Data

**DOI:** 10.3390/jcm13216522

**Published:** 2024-10-30

**Authors:** Ken Lund, Jan Nielsen, Simon Kjeldsen, Pedro Póvoa, Torben Knudsen, Bente Mertz Nørgård, Jens Kjeldsen

**Affiliations:** 1 Center for Clinical Epidemiology, Odense University Hospital, 5000 Odense, Denmark; 2 Department of Clinical Research, University of Southern Denmark, 5000 Odense, Denmark; jens.kjeldsen@rsyd.dk; 3 Department of Paediatrics and Adolescent Medicine, Rigshospitalet, 2100 Copenhagen, Denmark; 4 Intensive Care Unit 4, Department of Intensive Care, Hospital de São Francisco Xavier, ULSLO, Estrada do Forte do Alto do Duque, 1449-005 Lisbon, Portugal; 5 NOVA Medical School, CHRC, New University of Lisbon, Campo dos Mártires da Pátria, 1169-056 Lisbon, Portugal; 6 Department of Medicine, Hospital of Southwest Jutland, 6700 Esbjerg, Denmark; 7 Department of Regional Health Science, University of Southern Denmark, 5230 Odense, Denmark; 8 Department of Medical Gastrointestinal Diseases, Odense University Hospital, 5000 Odense, Denmark

**Keywords:** COVID-19, nationwide, comorbidity, hospitalization, mortality

## Abstract

**Objectives**: The influence of comorbidity on long-term hospitalization and mortality after COVID-19 in adults (40–59 years) and older adults (≥60 years) is yet to be explored. **Methods**: This is a Danish population-based cohort study of patients with a first-time positive PCR test for COVID-19 from 1 March 2020, to 28 February 2022 (N = 1,034,103). Exposed cohorts were patients with 1) a Charlson Comorbidity Index (CCI) score of 1–2 and 2) a CCI score ≥3, who were compared to patients without comorbidity (CCI of zero) within the groups of adults (67.9%) and older adults (32.1%) for the risk of hospitalization and mortality. Next, within the age groups, each disease category of the CCI was considered as an exposed cohort and compared to patients who did not have the specific disease of interest. Adjusted hazard ratios (HR) for hospitalization and mortality were estimated by Cox regression models adjusted for confounders. **Results**: The highest HRs were in adult patients with a CCI score of ≥3. The adjusted HR was 4.54 (95%CI: 4.38–4.70) for hospitalization, and among older adults it was 3.05 (95%CI: 2.99–3.11). The adjusted HR for mortality among adults with a CCI score ≥3 was 21.04 (95%CI: 18.86–23.47), and the adjusted HR for mortality among older adults was 4.61 (95%CI: 4.44–4.78). The underlying disease influenced the risk estimates among adults and older adults, and “dementia” had the highest impact on mortality. **Conclusion**: A CCI score of 1 or above increases the risk of hospitalization and mortality up to 2 years after a positive PCR test of COVID-19 for adults and older adults. Further, the type of underlying disease in older adults highly influences the risk of hospitalization and mortality.

## 1. Introduction

SARS-CoV-2 (severe acute respiratory syndrome coronavirus 2) infection, known as COVID-19, emerged in December 2019 in Wuhan, China, and spread around the world on an unprecedented scale [1,2]. In different study populations, the consequences of COVID-19 have been examined with a focus on short-term consequences [3,4,5,6].

The risk of infection by COVID-19 is present in both adults (40–59 years) and older adults (≥60 years), but the consequences differ by various predictors where the impact of comorbidity is an important focus area. Comorbidity can be present in all age groups, but in the general population, a higher age also often implies more comorbidity, which gives a special focus on the consequences for adults and older adults [7].

It is well known that age is an independent risk factor for a more serious disease course in the acute phase of the infection [3,4,6,8,9]. Other independent risk factors are male gender and comorbidities that negatively influence on outcomes after COVID-19 infections, i.e., increased severity, increased length of hospital stay, and mortality within short-term follow-up (30 days to 60 days) [3,4,10]. The number of comorbidities has also been shown to be a strong predictor of COVID-19 severity and death within 30 days [3].

It is not well explored to what extent comorbidity and age group may predict long-term adverse outcomes after COVID-19. Additionally, the impact of a single underlying disease on long-term adverse outcomes after COVID-19 in different age groups has not yet been assessed in a nationwide register-based study. Therefore, we aimed to examine the long-term impact of comorbidity in adults (40–59 years) and older adults (≥60 years) who have had a positive COVID-19 polymerase chain reaction (PCR) test, with respect to any hospitalization and mortality within 2 years.

## 2. Methods

### 2.1. Setting, Study Design, and Study Population

This is a Danish population-based cohort study. The study population included adults and older adults with an age above 40 years with a COVID-19 PCR test from 1 March 2020 to 28 February 2022. In Denmark, the health care system is tax-financed and with free access for all citizens. The Danish population is predominantly Caucasian (>90%) and there are approximately 5.9 million inhabitants. The uniform health care system and the use of nationwide registers with key information enables us to use an unselected nationwide study design [11,12]. We had access to the Danish Civil Registration System, the National Patient Register, and the nationwide COVID-19 surveillance database (PCR test results for COVID-19), which were made available by Statens Serum Institut to Danish researchers and stored in the Danish Microbiology Database [11,13,14,15]. In Denmark, the COVID-19 pandemic began at the end of February 2020, and community transmission of COVID-19 was present by March 2020 [5].

### 2.2. Data Sources

The Danish Departments of Clinical Microbiology and Statens Serum Institut carried out laboratory analysis, registration, and release of the national SARS-CoV-2 surveillance data for the present study. The surveillance data include all first-time positive PCR tests for COVID-19 and the personal identification number. Only data on the first positive PCR test for each individual are available. Data on vaccinations include the date of the first, second, and third vaccination and the brand used. The National Patient Register was established in 1977 and holds information from all hospitals on discharges and information on outpatient visits since 1994 [11]. The National Patient Register holds information on the personal identification number, the date of admission plus discharge, and procedure codes for in-hospital treatments. Discharge diagnoses are classified using the International Classification of Disease system (Version 8 was used before 1994, and version 10 after 1994). The Danish Civil Registration System was established in 1968 for all persons living in Denmark and includes key information such as personal identification number, sex at birth, date of birth, immigration, and mortality [14,15]. Data linkage between the registers with COVID-19 surveillance data, COVID-19 vaccination data, the National Patient Register, and the Danish Civil Registration System is possible at an individual level using the unique personal identification number given to all Danish citizens at birth.

## 3. Cohorts, Exposed and Unexposed

The study population was divided into two age groups, adults (40–59 years) and older adults (≥60 years), and examined separately for the impact of comorbidity in COVID-19 PCR-positive individuals. We used the Charlson Comorbidity Index (CCI) with 10 years of medical history (calculated backward from the date of the first PCR test) [16]. We chose the CCI as the prediction ability has been found appropriate in a Danish setting [17]. We used two different strategies to examine the influence of comorbidity. Firstly, within each age group, we created two separate exposed cohorts of patients with (1) a CCI score of 1–2 and (2) a CCI score ≥3. In both age groups, the reference groups were patients with a CCI score of null. Secondly, we examined the separate effects of each of the underlying diseases included in the CCI. Each specific disease category of the CCI was considered as a separate exposed cohort (in total nine exposed cohorts, as we merged three of the disease categories due to low numbers), and the reference group was patients with a CCI score of null. In these analyses, a patient may be represented in more than one disease category in these comparisons. The CCI was used as the prognostic value of the chronic diseases changed over time. These selected diseases are chosen based on one version of the CCI, and represent the most important non-malignant disease categories that have been shown to have significant prognostic value (i.e., assigned a weight of ≥1, congestive heart failure, dementia, chronic pulmonary disease, rheumatologic disease, mild liver disease, diabetes with chronic complications, hemiplegia or paraplegia, renal disease, and moderate or severe liver disease) [18].

### 3.1. Outcomes

The outcomes were any kind of hospitalization and mortality with 2 years of follow-up. Any hospitalization (yes/no) was retrieved from the National Patient Register. Any hospitalization was registered and the duration of admission was defined as a minimum of 12 h. We used the first hospitalization after the first positive COVID-19 test in case of multiple hospitalizations. Information on mortality was retrieved from the Danish Civil Registration System.

### 3.2. Statistics and Confounders

We tabulated baseline data of the age groups, adults and older adults, reporting the frequency and percentages of the main variables. Initially, within each age group, we estimated the hazard ratio (HR) of the outcomes in the groups with a CCI score of 3 or more versus those with no comorbidity (CCI score of zero), and those with a CCI score of 1–2 to those with no comorbidity. We used Cox regression models (multivariable) to compute the crude HR and adjusted HR (aHR) for any hospitalization and mortality. In the analysis for hospitalization, we adjusted the models for sex, age as a continuous variable, and number of vaccinations. In the analysis of mortality, we adjusted for sex, age as a continuous variable, number of vaccinations, and hospitalization as a time-varying variable. Next, we examined the impact of a specific single underlying disease category according to the CCI. We compared each disease category of the CCI to those without the specific disease of interest in a univariable model, i.e., those identified with congestive heart failure were compared to those without congestive heart failure. In these analyses, we adjusted for other comorbid diseases from the CCI, sex, age as a continuous variable, number of vaccinations, and hospitalization in a multivariable model. In the analysis of hospitalization we did not include hospitalization. A patient represented in more than one CCI category was eligible for each of the individual comparisons.

In a sensitivity analysis, according to the analysis of mortality, we tried to omit hospitalization as a time-varying variable from the regression model. In another sensitivity we examined the impact of CCI categories of 0, 1, 2, 3, and ≥4, and in a final analysis of mortality and hospitalization we included age as cubic splines in the model.

We retrieved information on sex and age at the time of a positive COVID-19 PCR test from the National Patient Register and the Danish Civil Registration System. In addition, we retrieved information on the type of hospitalization to the intensive care unit within 7 days after the PCR-positive test, but the variable was solely included for descriptive purposes and not used as a confounder. We retrieved information from the national SARS-CoV-2 surveillance data on the number of vaccinations. All confounders were selected a priori.

### 3.3. Approval and Ethics

This nationwide register study was approved by the Danish Data Protection Agency under the joint notification of the Region of Southern Denmark (Journal no.: 20/21823). Data were accessed through a secure server at the Danish Health Authorities. Studies based on register-based data that do not involve any direct contact with patients do not require ethical approval according to Danish law.

## 4. Results

In this cohort study, we included 1,034,103 persons with a positive PCR test, where 702,081 (67.9%) were adults aged 40–59 years and 332,022 (32.1%) were older adults aged ≥60 years. In the adult group, 371,718 (52.9%) were females, and 171,859 (51.8%) among older adults were females. In the adult group, 607,824 (86.6%) had no registered comorbidity according to the CCI, and among older adults, 202,773 (61.1%) had no comorbidity. The most predominant category of disease from the CCI was rheumatologic disease with 10,657 (1.5%) in the adult group, and likewise among older adults with 12,206 (3.7%) persons. Within 7 days after the first positive PCR test, 2559 (0.4%) of the adults and 9035 (2.7%) of the older adults were hospitalized. Table 1 describes the basic characteristics of the study population.

### 4.1. CCI Score and the Risk of Hospitalization and Mortality Within 2 Years

**Figure 1:**
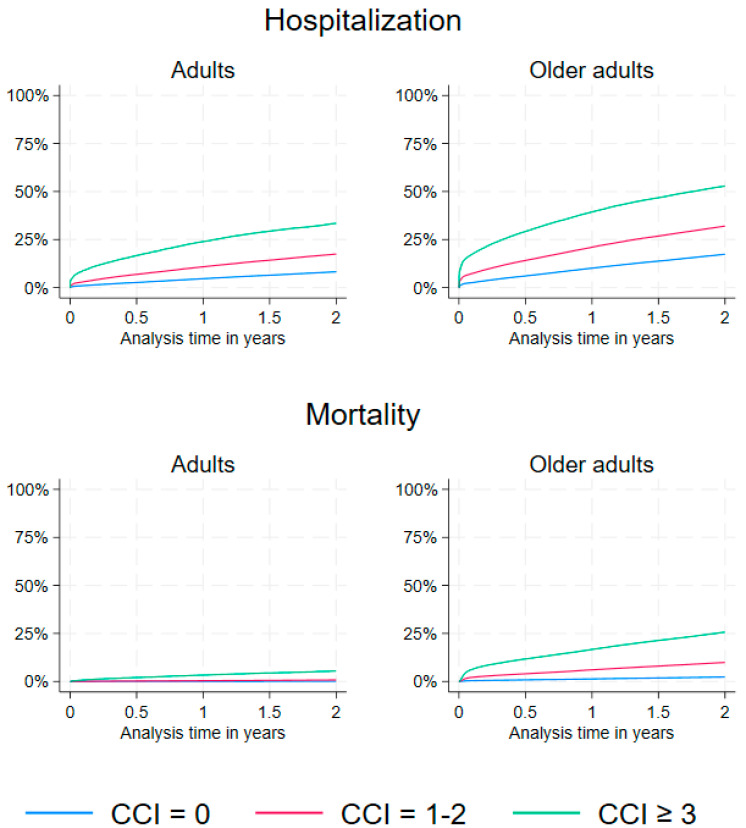
A panel of the cumulative incidence percentage of hospitalization (any kind) and mortality according to CCI score by adults (40–59 years, **left panel**) and older adults (≥60 years, **right panel**) in Danish PCR-positive COVID-19 patients with 2 years of follow-up.

The percentage of hospitalization within 2 years after a positive COVID-19 PCR test among adults increased with a higher CCI score. Specifically, hospitalization occurred in 8.2% of adults with a CCI score of 0, 17.4% of those with a CCI score of 1–2, and 33.3% of those with a CCI score of ≥3 (Table 2, Figure 1). Likewise, hospitalizations occurred in 17.3%, 31.4%, and 51.1% among older adults with CCI scores 0, 1–2, and ≥3, respectively (Table 2, Figure 1). The risk of hospitalization was increased in the CCI score 1–2 and ≥3 compared to persons without comorbidity in the adults and older adults group within 2 years of follow-up (Table 2). The highest adjusted risk estimate was 4.54 (95%CI: 4.38–4.70) in the adult group whereas the risk estimate in the older adult group was 3.05 (95%CI: 2.99–3.11) (Table 2).

Mortality in adults ranged from 0.2% in the group without comorbidity to 0.8% and 5.5% in the group with a CCI score of 1–2 or ≥3, respectively (Table 2, Figure 1). In older adults, the mortality was higher with 2.4% in the group without comorbidity, and 9.9% and 25.7% in the group with a CCI score of 1–2 or ≥3, respectively (Table 2, Figure 1). The adjusted risk of mortality for adults and older adults was increased depending on the CCI score categories 1–2 and ≥3 compared to persons with no comorbidity (Table 2). The highest adjusted risk of mortality was among adults with a CCI score ≥3 with 21.04 (95%CI: 18.86–23.47), and the risk of mortality among older adults was 4.61 (95%CI: 4.44–4.78) (Table 2). In the sensitivity analysis of mortality, where we omitted hospitalization from the model, the conclusions did not change.

### 4.2. CCI Disease Categories and Risk of Hospitalization and Mortality

**Figure 2:**
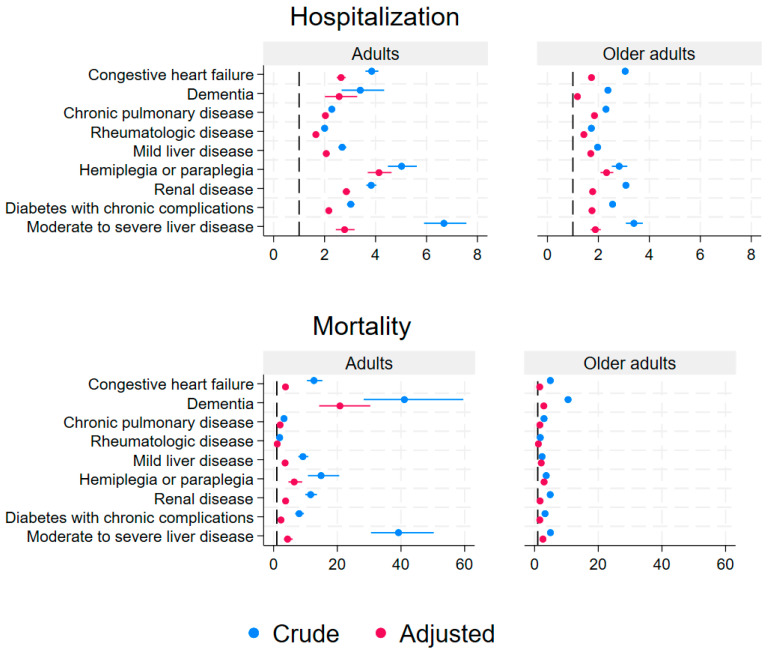
A panel of the crude and adjusted risk estimates for hospitalization and mortality by nine disease categories from the Charlson Comorbidity Index in adults (40–59 years, **left panel**) and older adults (≥60 years, **right panel**) with a PCR-positive COVID-19 test in Denmark with 2 years of follow-up.

Figure 2 shows the crude and adjusted risk estimates for hospitalization and mortality by disease categories from the CCI for adults and older adults. In adults, the three highest adjusted risk estimates for hospitalization were patients with hemiplegia or paraplegia with a HR of 4.13 (95%CI: 3.69–4.63), renal disease with a HR of 2.85 (95%CI: 2.71–3.01), and moderate or severe liver disease with a HR of 2.78 (95%CI: 2.44–3.18) (Figure 2, Supplementary Table S1). In older adults, the three highest adjusted risk estimates for hospitalization were patients with hemiplegia or paraplegia with a HR of 2.32 (95%CI: 2.08–2.58), and moderate or severe liver disease with a HR of 1.88 (95%CI: 1.69–2.09), and chronic pulmonary disease with a HR of 1.84 (95%CI: 1.81–1.88) (Figure 2, Supplementary Table S1).

All adjusted risk estimates for hospitalization depending on specific types of underlying disease categories among adults and older adults were statistically significantly increased, ranging from a HR of 1.18 (95%CI: 1.14–1.22) to 4.13 (95%CI: 3.69–4.63) (Figure 2, Supplementary Table S1). The type of underlying disease, associated with the highest proportions of hospitalization, was “moderate or severe liver disease” for adults at 46.7%, and in older adults it was also “moderate or severe liver disease” at 58.2%. For adults, the proportions of hospitalizations of different disease categories ranged from 18.2–46.7%, and in older adults, the proportions ranged from 37.4–58.2% (Supplementary Table S1).

All adjusted risk estimates for mortality in disease categories were statistically significantly increased in adults and older adults ranging from a HR of 1.61 (95%CI: 1.55–1.68) to 20.83 (95%CI: 14.28–30.37), except for adults with rheumatologic disease, where the HR was 1.13 (95%CI: 0.88–1.45) (Figure 2, Supplementary Table S1). The type of underlying disease with the highest proportions of mortality was “dementia” with 12.2% in adults and 47.6% in older adults (Supplementary Table S1). The percentage of mortality varied depending on disease categories from 0.6% to 12.2% in adults and from 11.7% to 47.6% in older adults (Supplementary Table S1). The sensitivity analysis, where we examined the impact of CCI categories of 0, 1, 2, 3, and ≥4, showed higher HRs with increasing CCI (Supplementary Table S2). Including age as cubic splines instead of a linear variable in the model did not change the results (Supplementary Table S2 and Figure S2).

## 5. Discussion

From 1 March 2020 to 28 February 2022 in this Danish nationwide cohort study, we identified 1,034,103 adults (40 years or older) with PCR-positive COVID-19, of which 32% were older adults aged ≥60 years. Hospitalization of any kind increased among adults and older adults with increasing CCI scores. In adults, the hospitalization rate ranged from 8.2% to 33.3%, and in older adults, it ranged from 17.3% to 51.1%, according to the cohort categorized by CCI. Comorbidity by the CCI was associated with an increased risk of hospitalization. Mortality increased with increasing CCI scores, regardless of age group. The mortality ranged from 0.2% to 5.5% in adults, and 2.4% in older adults to 25.7% according to the cohort categorized by CCI. We found that the risk of either outcome increased statistically significantly with increasing CCI scores in both adults and older adults, even in patients with a low CCI score of 1–2. When examining the different disease categories from the CCI in adults and older adults, the categories of “moderate or severe liver disease” were the most prognostic type of disease associated with hospitalizations. The risk of mortality was highest in the group of older adults with a CCI score of ≥3 compared to older adults without comorbidity, and “dementia” was the most frequent category for mortality for both age groups.

### 5.1. Hospitalization

The risk of any hospitalization was increased with increasing CCI category and the risk was increased for all disease categories, especially “dementia” and “renal disease”. The increased risk of hospitalization of our study was similar to the short-term increased risk reported by Reilev et al. [5], and in alignment with the observation of more frequent comorbidities among COVID-19 PCR-positive hospitalized patients [5]. Similar to Reilev et al. [5], the risk for any hospitalization was increased in all categories in our study, and this influence of age was also found in previous studies in other populations [19,20]. In addition, our study found that patients in the category “moderate or severe liver disease” were the ones with the most frequent hospitalizations.

### 5.2. Mortality

The risk of mortality after COVID-19 has been studied extensively on a short-term basis [6]. Several risk factors have been identified, such as age and comorbidity, and in our study, the risk of mortality was highest in the group of older adults with a CCI score of ≥3 compared to older adults without comorbidity. This finding extends our knowledge of the influence of comorbidity as a risk factor for mortality within 30 days, as found by Reilev et al. [5], where the number of comorbidities increased the risk of mortality and increasing age also increased the risk of mortality among Danish citizens. In a Canadian cohort, Ge et al. [3] have documented that pre-existing comorbidity was a prediction for 30-day mortality in individuals with COVID-19. Again, this study adds a long-term perspective on these associations when using the CCI as the exposure. Our results are not easily comparable with other studies because most studies have been focused on short-term outcomes. This is highlighted in a recent systematic review where an increasing number of diseases, a higher CCI, and an increased frailty level were shown to increase the likelihood of hospitalization and mortality, and where long-term outcomes were warranted [6]. Our study also showed that the risk for mortality was increased for nearly all categories of disease in older adults and adults, except for adult patients with “rheumatologic disease”. In a Swedish cohort of patients with COVID-19 requiring intensive care with one year of follow-up, mortality after 90 days was low, and males had an increased risk of mortality compared to women [21]. Exposure to COVID-19 in patients with comorbidities and long-term mortality could be related to several prognostic factors. Hägglöf et al. [21] found that several diseases, e.g., cardiac disease, COPD/asthma, diabetes, chronic liver disease, and chronic kidney disease were associated with higher mortality. The underlying mechanism for each of the factors may vary and be multifactorial as age also is a known risk factor for mortality in patients with COVID-19 [21,22]. Furthermore, patients with critical illnesses due to COVID-19 are perceived to have an increased risk of mortality later [23].

This study has several strengths. First, we used data from the Danish nationwide health registers, which are well-validated, and included data for the entire population, eliminating selection bias [11]. The unique data linkage between the Danish health registers and mandatory reporting to the registers also enables complete follow-up for all patients, which again eliminates selection bias. Secondly, the data on exposure are registered independently of the outcome data due to the prospectively mandatory reporting to the registers, reducing any information bias. The surveillance database has captured all PCR test results for COVID-19 since the beginning of the pandemic, the diagnosis in the Danish National Patient Register is based on ICD-10 coding, and extensive validation studies have been done [11]. Thirdly, our study adjusted for several important confounders such as sex, age, number of vaccinations, hospitalization, and sequelae of COVID-19 when estimating the long-term risk. Lastly, in Denmark, the COVID-19 pandemic began at the end of February 2020, and at the end of March 2020 the PCR test capacity for COVID-19 was up-scaled to include all individuals with mild to moderate respiratory symptoms suspicious of COVID-19 [5]. The extensive nationwide testing strategy used in Denmark reduced the risk of misclassification. Even though PCR has a high false-negative rate and can be biased towards specific groups, they are considered the best to detect COVID-19 [24].

The study is limited by only using the first available PCR test for COVID-19 for the patients and does not include repeated testing data. In this study, we focused on patients with a positive PCR test, and we were therefore not able to compare with “healthy patients” without COVID-19. The long-term consequences of COVID-19 were thus not compared with a non-COVID-19 cohort of healthy patients or patients with other acute infections to evaluate disease specific outcomes. In our analysis of mortality, we included hospitalization as a confounder. It could be argued that hospitalization is a mediator and not a confounder for these outcomes, but most often patients are hospitalized due to the severity of COVID-19 even though no exact threshold for hospitalization is available from an epidemiological point of view. By adjusting for hospitalization in our analysis we aim to take a proxy measure for the severity of COVID-19 into account. The focus on long-term outcomes for COVID-19 may introduce detection bias because of the introduction of home tests for COVID-19 in 2022, but in our study, we only included PCR-test data for COVID-19 until 28 February 2022, and hereby this bias should be of minor concern. Another limitation is that we have not included biomarkers and other relevant laboratory parameters, such as antinuclear antibodies (ANA) to COVID-19, which are prognostic factors for worse disease [25]. In patients with COVID-19, C-reactive protein levels and the neutrophil-to-lymphocyte ratio have been found to be a potential predictor of mortality [26]. The implication of these inflammatory biomarkers could potentially be examined in future studies focusing on patients with COVID-19 who experience delirium, where inflammation may have a significant role in the pathogenesis of delirium [27]. Additionally, we did not have data on whether patients were treated for their comorbid diseases and the severity of underlying disease, which could provide the basis for future additional analyses of the impact of comorbidity. When using observational register data for research, some inherent limitations are present and should be taken into consideration. This could be unmeasured or residual confounding, even though we were able to adjust for several confounders in our analyses. Furthermore, our data are not suitable to establish the underlying causes from exposure to COVID-19 to hospitalization or mortality, but are excellent for examining associations.

In conclusion, even minor comorbidity (a CCI score of 1–2) increases the risk of any type of hospitalization and mortality up to 2 years after a positive PCR test for COVID-19. There has been a general perception that high comorbidity is associated with worse COVID-19 outcomes, but our study shows that, regardless of age group, only mild comorbidity is associated with hospitalization and death. Furthermore, our study underlines that it is too simple just to refer to the importance of a high or low general status of comorbidity when considering long-term COVID-19 outcomes. In fact, our study shows that it is highly important to consider the impact of each individual’s specific type of underlying disease when it comes to COVID-19 outcomes. Some of the most serious types of underlying disease were hemiplegia or paraplegia, dementia, and moderate-severe liver disease. In this study, we have only examined the long-term risk of hospitalization and mortality after COVID-19. Future studies should examine other COVID-19 outcomes based on patients with certain risk profiles according to types of underlying diseases.

### 5.3. Patient and Public Involvement

We have presented the initial conceptualization and design of the study for patients and relative representatives who are a part of the research council at the Center for Clinical Epidemiology, Odense University Hospital. The representatives have contributed with input for the selection of the study population and ideas for choosing important outcomes. The representatives have not been involved in the conduct of the study.

## Figures and Tables

**Table 1:** Characteristic of Danish PCR-positive COVID-19 (first) according to adult (40–59 years) and older adult (≥60 years) patients from 1 March 2020 to 28 February 2022.

	Adults (40–59 y)	Older Adults (≥60 y)
Characteristic	n (%)	n (%)
N, Total	702,081	332,022
Age, median (25–75 percentiles)	49 (44–54)	68 (63–75)
Time of year for PCR test		
1 March 2020–31 August 2020	5629 (0.8)	4083 (1.2)
1 September 2020–28 February 2021	55,510 (7.9)	30,548 (9.2)
1 March 2021–31 August 2021	28,942 (4.1)	8481 (2.6)
1 September 2021–28 February 2022	612,000 (87.2)	288,910 (87.0)
Sex		
Female	371,718 (52.9)	171,859 (51.8)
Male	330,363 (47.1)	160,163 (48.2)
Charlson Comorbidity Index score ^a^		
No comorbidity (CCI = 0)	607,824 (86.6)	202,773 (61.1)
Intermediate (CCI = 1–2)	83,670 (11.9)	95,543 (28.8)
High (CCI = ≥3)	10,587 (1.5)	33,706 (10.2)
Charlson Comorbidity Index (CCI) category ^b^		
Congestive heart failure	2961 (0.4)	11,088 (3.3)
Dementia	230 (0.0)	8730 (2.6)
Chronic pulmonary disease	23,126 (3.3)	24,661 (7.4)
Connective tissue disease	10,657 (1.5)	12,206 (3.7)
Mild liver disease	5042 (0.7)	3262 (1.0)
Hemiplegia	804 (0.1)	684 (0.2)
Moderate-to-severe renal disease	4935 (0.7)	9547 (2.9)
Diabetes with end organ damage, Type 1 + 2	5974 (0.9)	9538 (2.9)
Moderate-to-severe liver disease	560 (0.1)	745 (0.2)
Type of hospitalization within 7 days after PCR-positive test		
ICU admission	232 (0.0)	730 (0.2)
Other admission	2559 (0.4)	9035 (2.7)

^a^ Charlson Comorbidity Index (CCI) calculated using 10 years of health data prior to the first positive PCR test. ^b^ A patient may be represented in more than one category in the Charlson Comorbidity Index.

**Table 2:** Outcomes according to CCI score in Danish PCR-positive COVID-19 (first) patients by adults (40–59 years) and older adults (≥60 years).

			Hazard Ratio	
Group/Outcome	Events n (%)	Time at Risk in Years	Crude HR (95% CI)	Adjusted ^a^ HR (95% CI)
Adults (40–59 y): Any hospitalization, 2 years				
CCI = 0 ^b^	49,868 (8.2)	1,155,552.6	1	1
CCI = 1–2	14,515 (17.4)	148,657.6	2.25 (2.21–2.29)	2.14 (2.10–2.18)
CCI ≥ 3	3444 (33.3)	15,903.9	4.90 (4.74–5.08)	4.54 (4.38–4.70)
Older (≥60 y): Any hospitalization, 2 years				
CCI = 0	34,814 (17.3)	360,562.3	1	1
CCI = 1–2	29,255 (31.4)	144,905.6	2.04 (2.01–2.08)	1.73 (1.70–1.76)
CCI ≥ 3	16,100 (51.1)	37,494.5	4.14 (4.07–4.22)	3.05 (2.99–3.11)
Adults (40–59 y): Mortality, 2 years				
CCI = 0	985 (0.2)	1,211,137.0	1	1
CCI = 1–2	689 (0.8)	166,273.2	5.09 (4.62–5.62)	4.20 (3.80–4.63)
CCI ≥ 3	578 (5.5)	20,447.8	34.72 (31.33–38.48)	21.04 (18.86–23.47)
Older (≥60 y): Mortality, 2 years				
CCI = 0	4810 (2.4)	399,619.1	1	1
CCI = 1–2	9473 (9.9)	179,494.4	4.35 (4.20–4.50)	2.43 (2.35–2.52)
CCI ≥ 3	8656 (25.7)	56,379.5	12.42 (11.99–12.86)	4.61 (4.44–4.78)

^a^ The models are adjusted for sex, age (continuous), number of vaccinations, and hospitalization (time-varying, but the variable is not included in the analysis of hospitalization).^b^ CCI: Charlson Comorbidity Index Score.

## Data Availability

The Danish nationwide data used in this study that supports the findings are accessible in raw format, and can be requested from the Danish Health Data Authority (kontakt@sundhedsdata.dk). The acquisition of data is limited and an application for an individual license for a research project is required. The authors do not have special privileges for acquiring the data.

## Supplementary Materials

The following supporting information can be downloaded at: https://www.mdpi.com/article/10.3390/jcm13216522/s1. Table S1. Outcomes according to CCI category in Danish PCR-positive COVID-19 (first) by Adults (40–59 years) and older adults (≥60 years) patients. Table S2. Sensitivity. Outcomes according to CCI score in Danish PCR-positive COVID-19 (first) patients by adults (40–59 years) and older adults (≥60 years), when using the age variable as a linear and with cubic splines. Figure S1. Sensitivity. A panel of the crude and adjusted risk estimates for hospitalization and mortality by 9 disease categories from the Charlson comorbidity index in adults (40–59 years, left panel) and older adults (≥60 years, right panel) with a PCR-positive COVID-19 test in Denmark with 2 years of follow-up, when using the age variable as a linear and with cubic splines.
