# Supplementary material for: The Impact of Comorbidity and Age on the Risk of Hospitalization and Mortality in Patients with Previous COVID-19 Infection—Based on Nationwide Data
Source: J Clin Med. 2024 Oct 30;13(21):6522. doi: 10.3390/jcm13216522 (PMC11546196; doi:10.3390/jcm13216522)
Supplement: Supplementary file 1 [file jcm-13-06522-s001.zip › jcm-3235780-supplementary.pdf]

Supplementary Tables for *"The impact of comorbidity and age on the risk of hospitalization and mortality in patients with previous COVID-19 infection - based on nationwide data"*

Supplementary Table S1. Outcomes according to CCI category in Danish PCR-positive COVID-19 (first) by Adults (40-59 years) and older adults (≥60 years) patients

	Exposed		Unexposed		Hazard ratios	
Group / Charlson Comorbidity Index category	Events n (%)	Time at risk in years	Events n (%)	Time at risk in years	Crude HR (95% CI)	Adjusted HR (95% CI) [a]
Adults (40-59y): Hospitalisation, 2 years						
Congestive heart failure	909 (31.4)	4,550.5	66,918 (9.6)	1,315,563.7	3.85 (3.61-4.11)	2.65 (2.48-2.83)
Dementia	65 (28.4)	364.8	67,762 (9.7)	1,319,749.3	3.40 (2.67-4.34)	2.57 (2.02-3.28)
Chronic pulmonary disease	4,563 (19.9)	40,096.3	63,264 (9.3)	1,280,017.8	2.28 (2.22-2.35)	2.03 (1.97-2.09)
Rheumatologic disease	1,927 (18.2)	18,890.2	65,900 (9.6)	1,301,224.0	2.00 (1.91-2.09)	1.66 (1.58-1.74)
Mild liver disease	1,157 (23.3)	8,364.0	66,670 (9.6)	1,311,750.1	2.69 (2.54-2.85)	2.07 (1.94-2.20)
Hemiplegia or paraplegia	304 (38.7)	1,148.6	67,523 (9.6)	1,318,965.5	5.02 (4.49-5.62)	4.13 (3.69-4.63)
Renal disease	1,497 (31.1)	7,588.3	66,330 (9.5)	1,312,525.8	3.83 (3.64-4.03)	2.85 (2.71-3.01)
Diabetes with chronic complications	1,515 (25.8)	9,755.2	66,312 (9.5)	1,310,358.9	3.03 (2.88-3.18)	2.17 (2.05-2.28)
Moderate or severe liver disease	250 (46.7)	699.6	67,577 (9.7)	1,319,414.5	6.68 (5.90-7.57)	2.78 (2.44-3.18)
Older (≥60y): Hospitalisation, 2 years						
Congestive heart failure	5,448 (53.0)	11,832.1	74,721 (23.7)	531,130.2	3.05 (2.97-3.14)	1.73 (1.68-1.78)
Dementia	3,427 (41.3)	9,211.1	76,742 (24.2)	533,751.3	2.37 (2.29-2.46)	1.18 (1.14-1.22)
Chronic pulmonary disease	10,276 (44.1)	31,439.8	69,893 (23.1)	511,522.5	2.30 (2.25-2.35)	1.84 (1.81-1.88)
Rheumatologic disease	4,399 (37.4)	17,290.4	75,770 (24.1)	525,672.0	1.72 (1.67-1.78)	1.43 (1.39-1.47)

Mild liver disease	1,297 (41.5)	4,351.1	78,872 (24.4)	538,611.3	1.97 (1.87-2.08)	1.70 (1.60-1.81)
Hemiplegia or paraplegia	333 (52.1)	750.6	79,836 (24.6)	542,211.8	2.82 (2.53-3.14)	2.32 (2.08-2.58)
Renal disease	4,705 (53.3)	10,053.7	75,464 (23.8)	532,908.7	3.08 (2.99-3.17)	1.77 (1.72-1.83)
Diabetes with chronic complications	4,299 (48.3)	11,195.3	75,870 (23.9)	531,767.1	2.56 (2.48-2.64)	1.75 (1.69-1.81)
Moderate or severe liver disease	406 (58.2)	746.5	79,763 (24.5)	542,215.9	3.39 (3.08-3.74)	1.88 (1.69-2.09)
Adults (40-59y): Mortality, 2 years						
Congestive heart failure	113 (3.8)	5,792.3	2,145 (0.3)	1,393,019.7	12.66 (10.48-15.30)	3.73 (3.05-4.56)
Dementia	28 (12.2)	426.8	2,230 (0.3)	1,398,385.2	41.06 (28.28-59.60)	20.83 (14.28-30.37)
Chronic pulmonary disease	225 (1.0)	45,943.3	2,033 (0.3)	1,352,868.7	3.26 (2.84-3.74)	2.04 (1.77-2.35)
Rheumatologic disease	64 (0.6)	21,224.6	2,194 (0.3)	1,377,587.4	1.89 (1.48-2.43)	1.13 (0.88-1.45)
Mild liver disease	139 (2.8)	9,903.8	2,119 (0.3)	1,388,908.1	9.20 (7.75-10.92)	3.60 (2.90-4.46)
Hemiplegia or paraplegia	37 (4.6)	1,561.6	2,221 (0.3)	1,397,250.3	14.89 (10.76-20.61)	6.46 (4.65-8.98)
Renal disease	169 (3.4)	9,660.3	2,089 (0.3)	1,389,151.6	11.63 (9.94-13.60)	3.77 (3.17-4.48)
Diabetes with chronic complications	144 (2.4)	11,767.6	2,114 (0.3)	1,387,044.4	8.03 (6.78-9.50)	2.32 (1.93-2.79)
Moderate or severe liver disease	64 (11.4)	1,037.0	2,194 (0.3)	1,397,775.0	39.22 (30.59-50.28)	4.38 (3.21-5.97)
Older (≥60 y): Mortality, 2 years						
Congestive heart failure	2,999 (27.0)	18,296.3	19,959 (6.2)	617,619.4	4.94 (4.76-5.14)	1.61 (1.55-1.68)
Dementia	4,153 (47.6)	12,393.6	18,805 (5.8)	623,522.1	10.52 (10.17-10.88)	2.90 (2.80-3.00)
Chronic pulmonary disease	4,193 (17.0)	44,065.2	18,765 (6.1)	591,850.5	2.96 (2.87-3.06)	1.67 (1.61-1.73)
Rheumatologic disease	1,430 (11.7)	22,629.1	21,528 (6.7)	613,286.6	1.79 (1.70-1.89)	1.20 (1.13-1.26)
Mild liver disease	499 (15.3)	5,895.4	22,459 (6.8)	630,020.3	2.35 (2.15-2.57)	2.09 (1.89-2.31)
Hemiplegia or paraplegia	158 (23.1)	1,181.3	22,800 (6.9)	634,734.4	3.65 (3.13-4.27)	2.98 (2.55-3.49)
Renal disease	2,592 (27.1)	15,681.1	20,366 (6.3)	620,234.6	4.90 (4.71-5.11)	1.72 (1.65-1.80)
Diabetes with chronic complications	1,890 (19.8)	16,642.8	21,068 (6.5)	619,272.9	3.29 (3.14-3.44)	1.64 (1.57-1.73)
Moderate or severe liver disease	222 (29.8)	1,205.6	22,736 (6.9)	634,710.1	4.99 (4.37-5.69)	2.62 (2.26-3.04)

[a] The models are adjusted for sex, age (continuous), number of vaccinations, hospitalization due to COVID-19 (time-varying, but the variable not is included in the analysis of hospitalization), and for concomitant comorbid disease category from the CCI.

Supplementary Table S2, Sensitivity. Outcomes according to CCI score in Danish PCR-positive COVID-19 (first) patients by adults (40-59 years) and older adults (≥60 years), when using the age variable as a linear and with cubic splines

Group / outcome	Events n (%)	Time at risk in years	Hazard ratio Crude HR (95% CI)	Adjusted [a] HR (95% CI)	Adjusted [b] HR (95% CI)
Adults (40-59 y): Any hospitalization, 2 years					
CCI=0 [c]	49,868 (8.2)	1,155,552.6	1	1	1
CCI=1	8,686 (16.5)	94,593.5	2.11 (2.07-2.16)	2.03 (1.98-2.07)	2.02 (1.98-2.07)
CCI=2	5,829 (19.0)	54,064.1	2.48 (2.41-2.55)	2.32 (2.26-2.39)	2.32 (2.26-2.38)
CCI=3	1,621 (30.9)	8,325.2	4.42 (4.21-4.65)	4.06 (3.87-4.27)	4.05 (3.85-4.25)
CCI≥4	1,823 (35.8)	7,578.7	5.43 (5.18-5.69)	5.06 (4.83-5.30)	5.05 (4.82-5.29)
Older (≥60 y): Any hospitalization, 2 years					
CCI=0	34,814 (17.3)	360,562.3	1	1	1
CCI=1	15,220 (30.5)	78,631.9	1.96 (1.93-2.00)	1.68 (1.65-1.71)	1.68 (1.65-1.71)
CCI=2	14,035 (32.6)	66,273.6	2.14 (2.10-2.18)	1.79 (1.75-1.82)	1.79 (1.75-1.82)
CCI=3	7,039 (44.6)	20,718.6	3.34 (3.25-3.42)	2.48 (2.42-2.55)	2.48 (2.42-2.55)
CCI≥4	9,061 (57.5)	16,775.9	5.10 (4.98-5.22)	3.71 (3.62-3.80)	3.71 (3.62-3.80)
Adults (40-59 y): Mortality, 2 years					
CCI=0	985 (0.2)	1,211,137.0	1	1	1
CCI=1	247 (0.5)	105,058.0	2.89 (2.51-3.32)	2.44 (2.12-2.81)	2.44 (2.12-2.81)
CCI=2	442 (1.4)	61,215.2	8.88 (7.93-9.93)	7.13 (6.36-7.99)	7.12 (6.35-7.98)
CCI=3	158 (3.0)	10,474.4	18.54 (15.67-21.93)	12.04 (10.15-14.29)	12.03 (10.14-14.28)
CCI≥4	420 (8.0)	9,973.4	51.69 (46.11-57.95)	30.63 (27.14-34.57)	30.62 (27.12-34.56)
Older (≥60 y): Mortality, 2 years					
CCI=0	4,810 (2.4)	399,619.1	1	1	1
CCI=1	4,753 (9.3)	96,666.5	4.06 (3.90-4.22)	2.31 (2.22-2.41)	2.33 (2.23-2.42)
CCI=2	4,720 (10.7)	82,827.9	4.69 (4.51-4.89)	2.56 (2.46-2.67)	2.59 (2.48-2.69)
CCI=3	3,332 (20.1)	29,051.7	9.35 (8.94-9.77)	3.61 (3.45-3.78)	3.64 (3.48-3.81)
CCI≥4	5,324 (31.1)	27,327.8	15.64 (15.04-16.26)	5.60 (5.38-5.83)	5.66 (5.44-5.90)

[a] The models are adjusted for sex, age (continuous as linear), number of vaccinations, and hospitalization (time-varying, but the variable was not included in the analysis of hospitalization).
[b] The models are adjusted for sex, age (continuous as cubic splines), number of vaccinations, and hospitalization (time-varying, but the variable was not included in the analysis of hospitalization).
[c] CCI: Charlson Comorbidity Index Score.

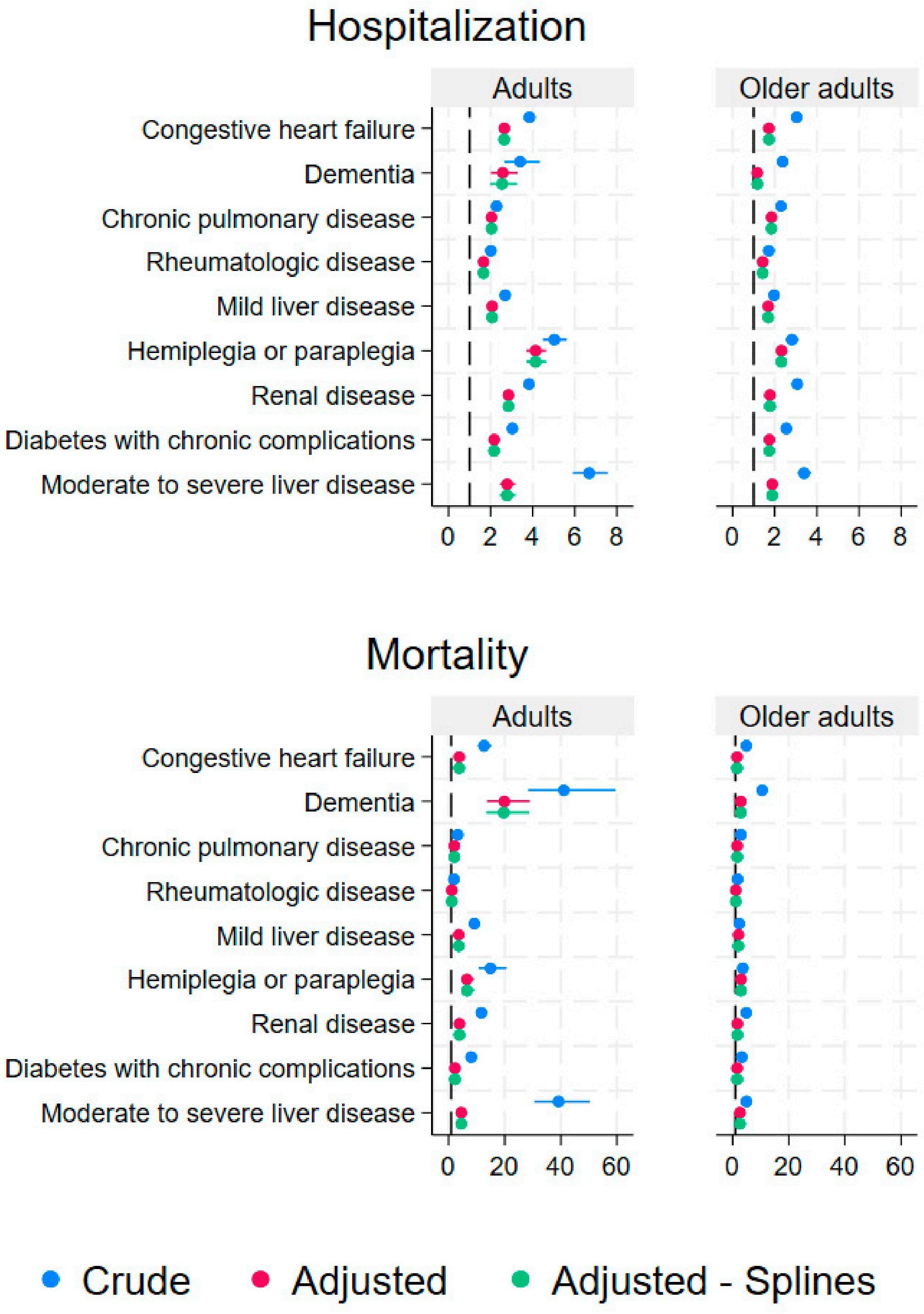

Supplementary Figure S1, Sensitivity. A panel of the crude and adjusted risk estimates for hospitalization and mortality by 9 disease categories from the Charlson comorbidity index in adults (40-59 years, left panel) and older adults (≥60 years, right panel) with a PCR-positive COVID-19 test in Denmark with 2 years of follow-up, when using the age variable as a linear and with cubic splines.
